# Identification of molecular alterations in leukocytes from gene expression profiles of peripheral whole blood of Alzheimer’s disease

**DOI:** 10.1038/s41598-017-13700-w

**Published:** 2017-10-25

**Authors:** Hongdong Li, Guini Hong, Mengna Lin, Yidan Shi, Lili Wang, Fengle Jiang, Fan Zhang, Yuhang Wang, Zheng Guo

**Affiliations:** 1 Department of bioinformatics, Key Laboratory of Ministry of Education for Gastrointestinal Cancer, Fujian Medical University, Fuzhou, China; 2 Fujian Key Laboratory of Tumor Microbiology, Fujian Medical University, Fuzhou, China

## Abstract

Blood-based test has been considered as a promising way to diagnose and study Alzheimer’s disease (AD). However, the changed proportions of the leukocytes under disease states could confound the aberrant expression signals observed in mixed-cell blood samples. We have previously proposed a method, Ref-REO, to detect the leukocyte specific expression alterations from mixed-cell blood samples. In this study, by applying Ref-REO, we detect 42 and 45 differentially expressed genes (DEGs) between AD and normal peripheral whole blood (PWB) samples in two datasets, respectively. These DEGs are mainly associated with AD-associated functions such as Wnt signaling pathways and mitochondrion dysfunctions. They are also reproducible in AD brain tissue, and tend to interact with the reported AD-associated biomarkers and overlap with targets of AD-associated PWB miRNAs. Moreover, they are closely associated with aging and have severer expression alterations in the younger adults with AD. Finally, diagnostic signatures are constructed from these leukocyte specific alterations, whose area under the curve (AUC) for predicting AD is higher than 0.73 in the two AD PWB datasets. In conclusion, gene expression alterations in leukocytes could be extracted from AD PWB samples, which are closely associated with AD progression, and used as a diagnostic signature of AD.

## Introduction

Alzheimer’s disease (AD) is the predominant form of dementia. The pathological features of AD include the presence of amyloid plaques, neurofibrillary tangles, synaptic loss, soluble amyloid-β (Aβ) oligomers, neuritic dystrophy, and eventual neurodegeneration^1^. In clinical practice, AD diagnosis is mainly based on PET imaging or cerebral spinal fluid biomarkers. The major disadvantages of these diagnostic approaches are the high cost, the low patient compliance, and most importantly, the difficulty in diagnosing AD at an early stage^2^.

The natural role of blood cells in immune response to physiologic and pathologic changes has made blood an important source for investigation of disease-associated molecular biomarkers^3^. Recent studies have also demonstrated a significant degree of covariability in gene expression between brain tissue and peripheral blood cells^4–7^. Therefore, a diagnostic blood biomarker for AD would be valuable and convenient for the early diagnosis of patients presenting at clinics with memory complaints. Actually, the potential use of peripheral whole blood (PWB) or peripheral blood mononuclear cell (PBMC) gene expression profiling in the diagnosis of brain disorders has been described^6–10^. These studies detected and analyzed the significantly altered genes^6,9^ or modules^8,10^ by directly comparing the expression measurements between AD and normal blood samples. It’s noted that relative proportions of the blood cells may shift under disease states^11,12^, which may confound the aberrant disease signals originated from leukocytes when directly comparing expression values of genes between disease and normal blood samples^13,14^. Consequently, the changed proportions of leukocyte subtypes could introduce some differentially expressed genes (DEGs) between disease samples and normal controls which actually have no expression changes in any leukocyte subtypes^15^. Therefore, it is necessary to exclude alteration signals originating from leukocyte subtype proportion changes when trying to detect AD-associated cellular molecular changes from mixed-cell blood samples.

In recent years, researchers have developed methods based on deconvolution^16^ or surrogate variable analysis algorithms^17,18^ to avoid the influence of relative leukocyte subtype proportion changes on the overall signals of PWB or PBMCs. Methods based on deconvolution algorithms aim to estimate and adjust the proportion of each leukocyte subtype in blood samples using the expression profiles of purified leukocyte subtypes^16^. However, the absolute quantitative gene expression level measurements used in these methods could be sensitive to systematic biases of microarray measurements especially examined in different microarray platforms^19^. Methods based on surrogate variable analysis aim to find true disease-associated alterations by estimating and adjusting the confounding factors that could have effects on gene expression levels^17,18^. However, it’s difficult for them to avoid the influence of cell proportion changes that are indeed associated with disease progression^15^. More recently, we proposed a method, *Ref-REO*, to detect leukocyte-specific molecular alterations from mixed-cell blood samples of patients through analyzing the disrupted patterns of the pre-determined within-sample relative expression orderings (REOs) of genes which are consistent in purified normal leukocyte subtypes^15^. This method is based on the fundamental that if the REOs of any two genes have consistent patterns (eg. ${E}_{A} > {E}_{B}$) in all normal leukocyte subtypes, these consistent patterns could be stable in PWB or PBMCs, no matter how the proportion of the constituent cells changes when no expression alterations occur in leukocytes. If inconsistent patterns are observed in disease samples, at least one of these two genes has altered gene expression in certain leukocyte subtypes. The *Ref-REO* method has been shown to have higher precision and recall than the previous methods^15^. Most importantly, the REOs of genes have been reported to be more robust than the absolute measured levels as REOs are invariant to monotonic data transformation (normalization) and rather resistant to batch effects^19,20^, indicating the disease-associated biomarkers detected by this method could be easily validated and transferred.

Therefore, in this study, we apply the *Ref-REO* method to detect and analyze the AD-associated cellular expression alterations from two independent AD PWB datasets. The results showed that these AD-associated molecular alterations detected from PWB by *Ref-REO* were significantly enriched in AD-associated pathways. They were reproducible in brain tissue of AD-patients, and had interactions with reported AD biomarkers and overlaps with the targets of AD-associated miRNAs.

## Materials and Methods

### Datasets

The gene expression data were downloaded from the Gene Expression Omnibus database (GEO, http://www.ncbi.nlm.nih.gov/geo/). Detailed information for each dataset was described in Table 1. The PLS-47 (GSE28490) dataset examined 47 expression profiles for nine leukocyte subtypes, which were isolated from healthy human blood and assessed for cell type purity by flow cytometry^21^. The PLS-33 (GSE28491) dataset examined 33 expression profiles for seven leukocyte subtypes, which were obtained from a separate panel of healthy donors at the University Hospital of Geneva. These two datasets were used to detect the gene pairs with stable REOs in each purified leukocytes^21^. The PWB-AD-249 (GSE63060) and PWB-AD-275 (GSE63061) datasets examined the PWB expression profiles for AD and normal control samples which were obtained from the AddNeuroMed consortium, a large cross-European AD biomarker study and a follow-on Dementia Case Register (DCR) cohort in London^22^. These two datasets were used to detect the AD-associated molecular alterations in leukocytes. The PWB-Normal-61 dataset included the PWB expression profiles for 61 healthy controls with age ranging from 18 to 56, which were obtained from the GEO dataset (GSE19151)^23^. In GSE19151, control samples of unknown age were excluded, thus only the 61 samples with age information were collected in PWB-Normal-61, which was used to detect aging-associated genes. The Brain-AD-161 (GSE5281) dataset examined 161 expression profiles of six brain regions for AD and normal control samples^24^. This dataset was used to evaluate whether the AD-associated PWB molecular alterations had expression changes in brain tissue. For each dataset, the normalized data were downloaded from GEO. The original platform annotation file obtained from GEO for each dataset was used to annotate the CloneIDs to GeneIDs. The number of genes measured in each dataset was shown in Table 1. Totally, 8,708 genes commonly measured in all datasets were analyzed in the study.

**Table 1:** Datasets analyzed in this study.

Dataset^*^	Characteristic of Sample	Age (Years)	Platform (#Gene)	GEO Accession ID	Ref
**Purified leukocyte subtypes (PLS)**					
PLS-47	All: 47 Monocytes: 10, B cells:5, CD4+ T cell:5, NK cells:5, CD8+ T cell:5, Eosinophils:4, mDCs:5; Neutrophils:3, pDCs:5	—	GPL570 (11,241)	GSE28490	21
PLC-33	All: 33 CD19+ B cells: 5, CD14+ monocytes:5, CD4+ T cells:5, CD8+ T cells:5, Eosinophils:3, NK cells:5; Neutrophils:5	—	GPL570 (10,689)	GSE28491	21
**peripheral whole blood (PWB)**					
PWB-AD-249	ALL:249 Control: 104; AD:145	52 ~ 90	GPL1122 (21067)	GSE63060	22
PWB-AD-275	ALL:275 Control: 135;AD:140	57 ~ 100	GPL1122 (18327)	GSE63061	22
PWB-Normal-61	61	18 ~ 56	GPL571 (12432)	GSE19151	23
**Brain tissue**					
Brain-AD-161	Brain region (Control: AD) Entorhinal Cortex (13:10) Hippocampus (13:10) Middle temporal gyrus (12:16) Posterior cingulate cortex (13:9) Superior frontal gyrus (11:23) Primary visual cortex Control (12:19)	63 ~ 102	GPL570 (20848)	GSE5281	24

^*^The abbreviation of each dataset is denoted by the phenotype followed by sample size.

### Detecting disease-associated cellular alterations from AD PWB samples

**Figure 1:**
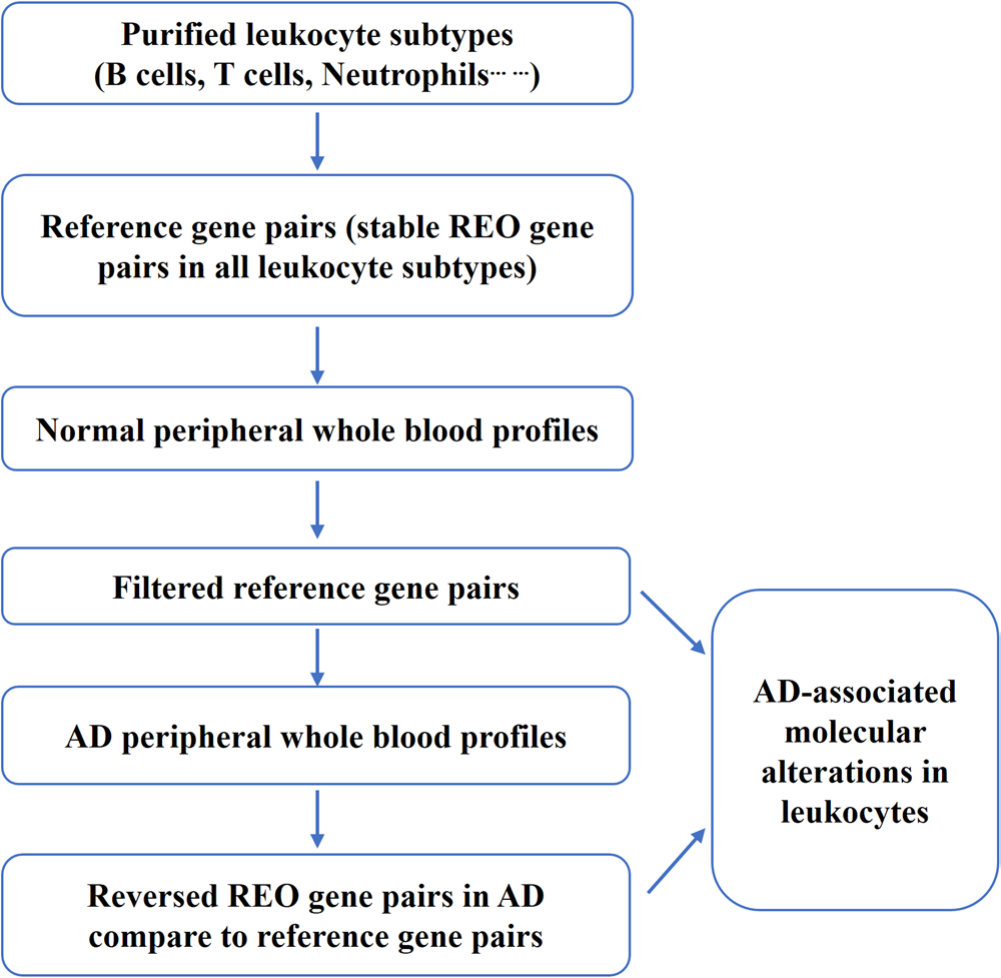
The flow chart of detecting AD-associated cellular alterations by the *Ref-REO* method.

The *Ref-REO* method was employed to detect the AD-associated cellular alterations from AD PWB samples^15^. Briefly, the algorithm detected the disease-associated cellular alterations according to the following steps as shown in Fig. 1: (1) Select reference gene pairs. Reference gene pairs are gene pairs whose REOs are stable and consistent across different purified normal leukocytes. The REOs of these gene pairs could be stable under normal condition or disease state, no matter how the proportion of the constituent cells changes when no expression alterations occur in leukocytes. (2) Filter the reference gene pairs by the control samples in the dataset under study to exclude the gene pairs whose REOs are easily affected by age, sex, experimental batch effects^25^ and other factors. (3) Detect reversed gene pairs. Reversed gene pairs are the gene pairs that have inconsistent REO patterns with reference gene pairs in the disease samples. The reversed REO patterns of these gene pairs should be caused by expression alterations occurred in leukocyte subtypes, given that the changed proportion of the constituent cells could not affect REOs of the reference gene pairs when no expression alterations occur in leukocytes. (4) Detect DEGs. Based on the filtered reference gene pairs and reversed gene pairs, whether a gene could be observed in the reversed gene pairs by random chance was evaluated by the hypergeometric distribution model. The DEGs were detected as significant if the adjusted *P*-value was less than 0.05^15^.

### Detecting DEGs in various brain regions of AD patients

The student’s *t*-test was used to detect DEGs in various brain regions of AD patients comparing to normal controls. P-values were adjusted for multiple testing using the Benjamini-Hochberg procedure to control the FDR level. If the adjusted p-value for a gene was less than 0.05, this gene was considered as a DEG.

### Detecting aging-associated genes from normal PWB samples

Because the relative proportions of the blood cells in older adults shifted weakly as age increases^12^, the linear regression model was employed to detect the aging-associated genes in the normal PWB controls of the datasets analyzed in this study. P-values were adjusted for multiple testing using the Benjamini-Hochberg procedure^26^ to control the FDR level at 0.05. If the adjusted p-value for a gene was less than 0.05, this gene was considered as an aging-associated gene.

### Random experiments

Two random experiments were performed in this study.

The first random experiment was performed to evaluate whether the number of reversed gene pairs detected in a dataset was significantly more than expected by chance. Supposed there are *n* reversed gene pairs observed in a study dataset. The random experiment consists of the following steps: (1) Randomly disturb sample labels. The sample sizes of normal controls and AD samples are kept the same in randomized data and in the study dataset. (2) Calculate the number of reversed gene pairs detected in the randomized data, denoted as *m*. (3) Repeat step 2 for 1,000 times and calculate the percentage of the random experiments in which *m* is larger than *n*, defined as the probability of observing *n* reversed gene pairs by random chance. A p-value <0.05 was considered as significant.

The second random experiment was performed to evaluate whether the number of observed AD-DEGs having interactions with the AD-associated biomarkers was significantly more than expected by chance. Suppose *k* out of *n* AD-DEGs interact with at least one of the AD-associated biomarkers, the random experiments were done with the following steps: (1) Randomly select *n* genes from the background genes as the randomized AD-DEGs. (2) Calculate the number of the randomly defined AD-DEGs that interact with at least one of the AD-associated biomarkers, denoted as *m*. (3) Repeat step 2 for 1,000 times and calculate the percentage of the random experiments in which *m* is larger than *k*, defined as the probability of observing *k* AD-DEGs interacting with at least one of AD-associated biomarkers by random chance. A p-value <0.05 was considered as significant.

### Enrichment analysis

The KEGG (Kyoto Encyclopedia of Genes and Genomes) and Gene Ontology databases were used to evaluate the AD-associated cellular alterations in PWB by the functional annotation tool DAVID (https://david.ncifcrf.gov/, version 6.8)^27^. For a given dataset, all of the measured genes annotated in the KEGG or Gene Ontology database were considered as the background genes.

### String, AlzGene, and miRTarBase databases

The protein-protein interactions were downloaded from STRING database (https://string-db.org/, Version 10) that collected known and predicted 82,160 protein-protein interactions involving 7,638 proteins^28^. The AD-associated biomarkers were download from AlzGene database which is a collection of published AD genetic association studies^29^ including 618 genes (http://alsgene.org/, download at April, 2017). These two databases were used to evaluate the interactions between AD-associated cellular alterations in PWB and AD-associated alterations collected in AlzGene database. MiRNA-mRNA interactions were downloaded from miRTarBase database at April 2017 (http://mirtarbase.mbc.nctu.edu.tw/). In this study, only the experimentally validated microRNA-target interactions were downloaded, which included 322,161 miRNA-mRNA interactions involving 2,618 miRNAs and 14,831 mRNAs^30^.

## Results

### AD-associated cellular alterations in PWB

First, gene pairs with stable REOs in different normal leukocytes were detected in purified leukocyte expression datasets using the *Ref-REO* method. Totally, 9,638,173 and 8,832,824 gene pairs were detected in the gene expression profiles of purified leukocyte subtypes examined in PLS-47 and PLS-33, respectively. The two lists shared 6,133,414 gene pairs, and 99.9% of them had consistent REO patterns, which was unlikely to happen by chance (binomial distribution test, p-value <2.2 × 10^−16^). These consistent gene pairs (totally 6,124,866) were used as the reference for recognition of abnormal disease signals in leukocytes.

Then, the reference gene pairs were evaluated in AD PWB expression profiles examined in PWB-AD-249 and PWB-AD-275, respectively. For dataset PWB-AD-249, 4,524,607 of the reference gene pairs retained the REO patterns in at least 95% of the 104 normal PWB profiles. Among them, 1,145 gene pairs had significantly reversed REO patterns in the 145 AD PWB profiles (Fisher exact test, adjusted p-value <0.05). For dataset PWB-AD-275, 4,673,704 genes pairs had consistent REO patterns with the reference in at least 95% of the 135 normal PWB profiles. Among them, 1,249 gene pairs showed significantly reversed REO patterns in the 140 AD PWB profiles (Fisher exact test, adjusted p-value <0.05). Though relatively little in quantity, these reversed gene pairs may reflect the true AD-associated information, as no reversed genes pairs could be detected in the 1,000 random experiments by randomly disturbing the sample labels of controls and cases (p-value <0.001, see Materials and Methods).

Based on the reference gene pairs and reversed gene pairs, DEGs were detected from PWB-AD-249 and PWB-AD-275 using the *Ref-REO* method^15^. Totally, with FDR <5%, 42 and 45 DEGs were detected respectively (Table 2), which shared 21 genes. All of the 21 shared DEGs had consistent expression dysregulated directions (up- or down-regulation) in AD samples compared to normal samples in the two datasets, which was unlikely to happen by chance (binomial distribution test, p-value <2.2 × 10^−16^). This result indicated the cellular alterations detected from AD PWB could be reproducible. In the following study, genes detected as significant in at least one of these two AD datasets were analyzed, denoted as AD-DEGs, which included 66 genes.

**Table 2:** DEGs detected from PWB-AD-249 and PWB-AD-275.

Gene ID	Symbol	Direction	P-value	Gene ID	Symbol	Direction	P-value
DEGs detected from PWB-AD-249				DEGs detected from PWB-AD-275			
5684	*PSMA3*	down	<2.2 × 10^−16^	2079	*ERH*	down	<2.2 × 10^−16^
9360	*PPIG*	down	<2.2 × 10^−16^	6160	*RPL31*	down	<2.2 × 10^−16^
51574	*LARP7*	down	<2.2 × 10^−16^	9552	*SPAG7*	down	<2.2 × 10^−16^
51611	*DPH5*	down	<2.2 × 10^−16^	51188	*SS18L2*	down	<2.2 × 10^−16^
84987	*COX14*	down	<2.2 × 10^−16^	5716	*PSMD10*	down	<2.2 × 10^−16^
7381	*UQCRB*	down	7.00 × 10^−9^	7155	*TOP2B*	down	1.35 × 10^−11^
6741	*SSB*	down	1.98 × 10^−8^	522	*ATP5J*	down	4.74 × 10^−11^
219927	*MRPL21*	down	3.83 × 10^−6^	20	*ABCA2*	up	2.32 × 10^−8^
57396	*CLK4*	down	4.04 × 10^−6^	5204	*PFDN5*	down	5.59 × 10^−8^
10600	*USP16*	down	1.83 × 10^−5^	79746	*ECHDC3*	up	5.69 × 10^−8^
51637	*C14orf166*	down	1.03 × 10^−4^	10632	*ATP5L*	down	6.84 × 10^−8^
11168	*PSIP1*	down	3.31 × 10^−4^	23500	*DAAM2*	up	1.85 × 10^−6^
126208	*ZNF787*	up	5.17 × 10^−4^	27089	*UQCRQ*	down	3.35 × 10^−4^
762	*CA4*	up	6.52 × 10^−4^	8664	*EIF3D*	down	6.21 × 10^−4^
23478	*SEC*. *11* *A*	down	3.00 × 10^−3^	5683	*PSMA2*	down	1.69 × 10^−3^
65056	*GPBP1*	down	3.32 × 10^−3^	51019	*CCDC53*	down	1.73 × 10^−3^
5685	*PSMA4*	down	1.20 × 10^−2^	29093	*MRPL22*	down	1.89 × 10^−3^
202018	*TAPT1*	down	1.51 × 10^−2^	9550	*ATP6V1G1*	down	2.56 × 10^−3^
84343	*HPS3*	up	1.55 × 10^−2^	6256	*RXRA*	up	4.15 × 10^−3^
6038	*RNASE4*	up	1.49 × 10^−2^	23294	*ANKS1A*	up	4.65 × 10^−3^
54556	*ING3*	down	4.01 × 10^−2^	4082	*MARCKS*	up	1.18 × 10^−3^
				85464	*SSH2*	up	1.66 × 10^−2^
				286006	*LSMEM1*	up	3.46 × 10^−2^
				22900	*CARD8*	up	4.26 × 10^−2^
**Overlapped DEGs between PWB-AD-249 and PWB-AD-275**							
521	*ATP5I*	down	<2.2 × 10^−16^	51371	*POMP*	down	<2.2 × 10^−16^
2959	*GTF2B*	down	<2.2 × 10^−16^	80135	*RPF1*	down	<2.2 × 10^−16^
3301	*DNAJA1*	down	<2.2 × 10^−16^	3476	*IGBP1*	down	9.67 × 10^−13^
4694	*NDUFA1*	down	<2.2 × 10^−16^	153527	*ZMAT2*	down	1.06 × 10^−11^
6119	*RPA3*	down	<2.2 × 10^−16^	8813	*DPM1*	down	2.22 × 10^−11^
6154	*RPL26*	down	<2.2 × 10^−16^	25847	*ANAPC13*	down	6.66 × 10^−9^
6233	*RPS27A*	down	<2.2 × 10^−16^	388789	*LINC00493*	down	1.12 × 10^−6^
9553	*MRPL33*	down	<2.2 × 10^−16^	1622	*DBI*	down	7.53 × 10^−6^
51258	*MRPL51*	down	<2.2 × 10^−16^	6645	*SNTB2*	up	3.35 × 10^−5^
51503	*CWC15*	down	<2.2 × 10^−16^	55505	*NOP10*	down	3.98 × 10^−3^
55854	*ZC3H15*	down	<2.2 × 10^−16^				

For the AD-DEGs, the enrichment analysis was conducted based on KEGG and Gene Ontology databases by DAVID^27^. The result showed that these DEGs were significantly enriched in oxidative phosphorylation, proteasome and ribosome pathways in KEGG, and Wnt signaling pathway and translation pathway in Gene Ontology (Table 3). These enriched pathways have been demonstrated to be closely associated with AD development and progression. For example, oxidative phosphorylation has been reported to be down-regulated in AD patients^31,32^. Ribosome dysfunction has been considered as an early event in AD progression^33^. The inhibition of proteasome activity has been reported to be sufficient to induce neuron degeneration in AD^34^. Specially, 10 AD-DEGs were enriched in mitochondrial inner membrane, suggesting that mitochondrial dysfunctions could play an important role in AD development. In fact, mitochondrial dysfunctions have been found in PWB lymphocytes of AD patients, which have been considered as a trigger of AD pathophysiology^35,36^.

**Table 3:** Pathways and functional terms enriched for AD-DEGs.

ID	Name	Hit Genes	P-value
**KEGG**			
hsa00190	Oxidative phosphorylation	*ATP5I*, *ATP5J*, *NDUFA1*, *UQCRB*, *ATP6V1G1*, *ATP5L*, *UQCRQ*	6.63 × 10^−5^
hsa03010	Ribosome	*RPL26*, *RPL31*, *RPS27A*, *MRPL21*, *MRPL22*, *MRPL33*	6.62 × 10^−4^
hsa03050	Proteasome	*PSMA2*, *PSMA3*, *PSMA4*, *POMP*	5.15 × 10^−3^
**Gene ontology**			
molecular function			
GO:0003735	structural constituent of ribosome	*RPL26*, *RPL31*, *RPS27A*, *MRPL21*, *MRPL22*, *MRPL33*, *MRPL51*	2.43 × 10^−4^
O:0003723	RNA binding	*RPF1*, *LARP7*, *RPL31*, *C14orf166*, *ZMAT2*, *RPL26*, *MRPL21*, *SSB*, *CWC15*, *EIF3D*	1.18 × 10^−3^
cellular component			
GO:0005743	mitochondrial inner membrane	*ATP5J*, *ATP5I*, *MRPL22*, *UQCRB*, *ATP5L*, *MRPL21*, *MRPL51*, *NDUFA1*, *UQCRQ*	2.25 × 10^−4^
GO:0019773	proteasome core complex,	*PSMA2*, *PSMA3*, *PSMA4*, *POMP*	8.89 × 10^−4^
biological process			
GO:0060071	Wnt signaling pathway	*PSMA2*, *PSMA3*, *PSMA4*, *RPS27A PSMD10*	9.50 × 10^−4^
GO:0006412	translation	*RPL26*, *RPL31*, *RPS27A*, *MRPL21*, *MRPL22*, *MRPL33*, *MRPL51*	3.57 × 10^−3^

### Expression changes of AD-associated PWB cellular alterations in various brain regions of AD patients

The Brain-AD-161 dataset, which examined expression profiles of six different brain regions in AD and normal controls, was used to evaluate the expression changes of PWB AD-DEGs in brain tissue of AD patients. By using the Student’s t-test with FDR <5%, the DEGs were detected from various brain regions of AD patients (Table 4). The AD-DEGs were found to significantly overlap with the DEGs detected from hippocampus, middle temporal gyrus, superior frontal gyrus and primary visual cortex of AD patients (Table 4). For example, among the 66 AD-DEGs, 53 DEGs were detected as DEGs in superior frontal gyrus of AD patients (hypergeometric distribution test, p-value = 7.30 × 10^−4^) and 49 out of them had consistent alteration directions in AD patients compared to normal controls. Totally, 60 out of the 66 AD-DEGs had expression changes in at least one of the six brain regions between AD and normal control samples, among which 52 genes had consistent alteration directions in PWB and brain tissue. The results further suggested that the cellular alterations observed in PWB could be closely associated with AD.

**Table 4:** Comparison of PWB AD-DEGs with DEGs detected from various brain regions of AD patients.

Brain region	DEGs	Overlapped with AD-DEG	P-value^*^
Entorhinal Cortex	1334	5 (5)^#^	0.98
hippocampus	2346	26 (21)	1.90 × 10^−2^
Middle temporal gyrus	2253	28 (26)	2.52 × 10^−3^
Posterior cingulate cortex	3273	24 (24)	0.64
Superior frontal gyrus	5344	53 (49)	7.3 × 10^−4^
Primary visual cortex	373	9 (9)	1.91 × 10^−3^

^#^The number outside the parentheses indicates the number of overlapped genes between AD-DEGs and brain tissue DEGs and the number inside the parentheses indicates the number of overlapped genes with consistent expression alterations in both PWB and brain tissue of AD patients. ^*^Represent the probability of observing the number of overlapped genes by chance calculated by the hypergeometric distribution model.

### AD-associated cellular alterations in PWB tend to interact with reported AD-biomarkers

**Figure 2:**
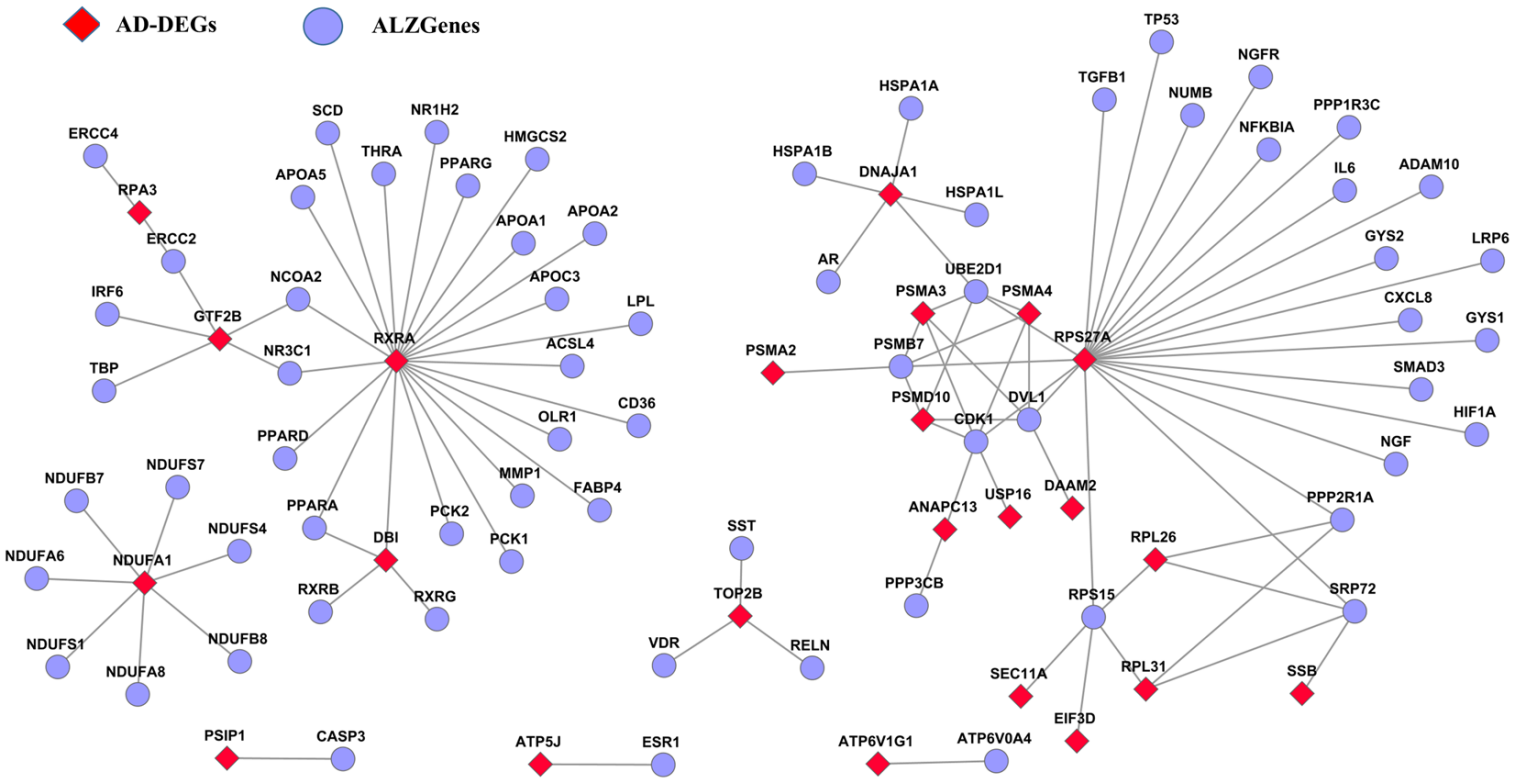
Interactions between AD-DEGs and AD-associated biomarkers.

The AD-DEGs were compared with the 618 AD-associated biomarkers collected from AlzGene database^29^. The result showed that only three AD-DEGs (ABCA2, CARD8 and RXRA) overlapped with the AD-associated biomarkers. With the protein-protein interaction data from STRING, we found 23 AD-DEGs each interacting with at least one of the AD-associated biomarkers (Fig. 2). The number was significantly more than expected by chance (p-value = 0.027): when randomly choosing the same number of genes as AD-DEGs, the mean number of random DEGs having interactions with the AD-associated biomarkers was 16.37 ± 3.6878, which was estimated in the 1,000 random experiments (see Materials and Methods). With FDR <0.05, the KEGG pathway enrichment analysis showed the interaction partners of AD-DEGs also tended to be enriched in the AD-associated oxidative phosphorylation (p-value = 5.2 × 10^−4^) and RRAR signaling pathways (p-value = 1.30 × 10^−6^)^37^.

**Figure 3:**
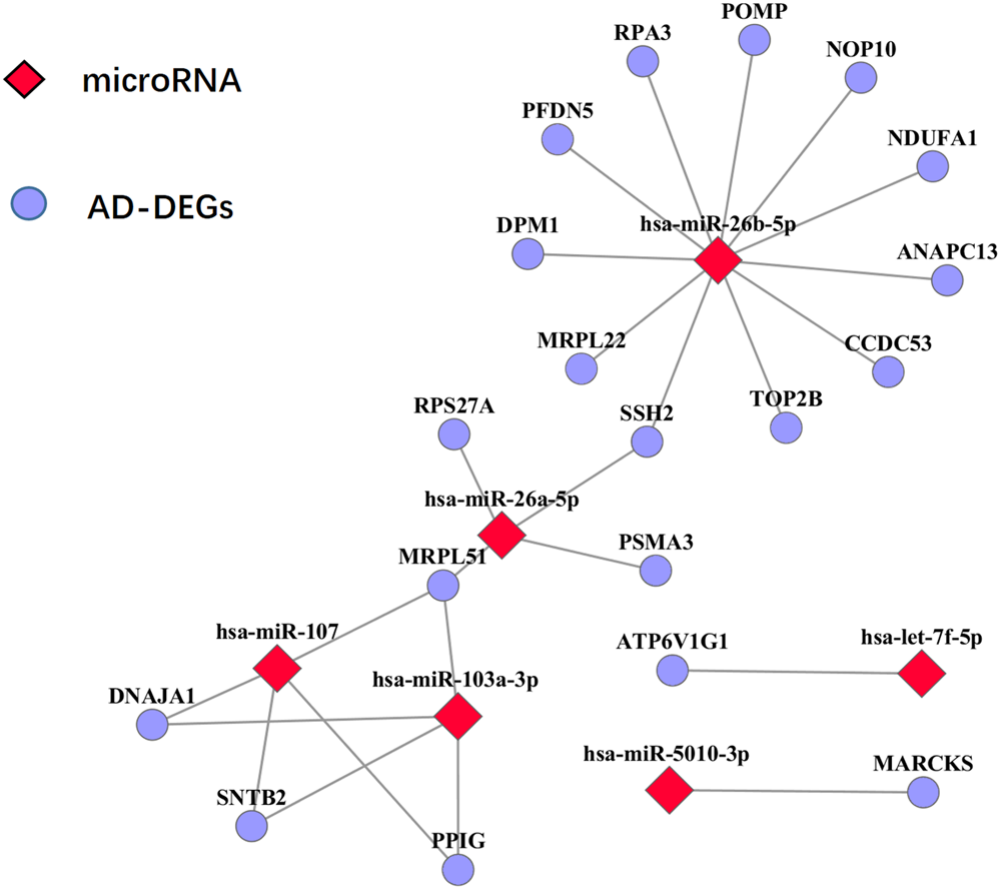
Interactions between AD-DEGs and PWB aberrant miRNAs.

AD-associated aberrant miRNA alterations observed in PWB were also analyzed for the AD-DEGs. The PWB aberrant miRNAs were collected from a study, which detected 12 diagnostic miRNAs (brain-miR-112, brain-miR-161, hsa-let-7d-3p, hsa-miR-5010-3p, hsa-miR-26a-5p, hsa-miR-1285-5p, hsa-miR-151a-3p, hsa-miR-103a-3p, hsa-miR-107, hsa-miR-532-5p, hsa-miR-26b-5p and hsa-let-7f-5p) between 48 AD patients and 22 non-AD control PWB samples^38^. From the miRTarBase database^30^, 1620 targets of the 10 miRNAs (two miRNAs weren’t collected by miTarbase) were downloaded. Among the 66 AD-DEGs, 19 were included in the 1620 targets, which was unlikely to happen by chance (hypergeometric distribution test, p-value = 0.028) (Fig. 3).

The above results further indicated that the AD-DEGs could be closely associated with AD development and progression.

### AD-associated cellular alterations in PWB and aging-associated genes

Aging-associated genes were detected from normal control samples in PWB-AD-249, PWB-AD-275 and PWB-Normal-61 using the linear regression model (see Materials and Methods). With FDR <5%, 1,332 and 3,146 aging-associated genes were identified in PWB-AD-249 and PWB-Normal-61, respectively, while no aging-associated genes were identified in PWB-AD-275. The numbers of detected aging-associated genes differed significantly in the three datasets, which could be explained by the different age distribution patterns: there were 104 normal controls with ages ranging from 52 to 87 in PWB-AD-249, while in PWB-AD-275, there were 135 normal controls with ages ranging from 63 to 91; and the PWB-AD-249 dataset had more samples with age ≤ 65 (9.62%) than the PWB-AD-275 dataset (2.22%, Fisher’s exact test, p-value = 0.019). In PWB-Normal-61, the age distribution was wider: there was 61 samples with ages ranging from 18 to 56. Compared the aging-associated genes identified in PWB-AD-249 and PWB-Normal-61, the results showed that these two lists shared 515 aging-associated genes (hypergeometric distribution test, p-value = 1.90 × 10^−2^), and 479 out of them had the same alteration directions with age in both datasets, which was unlikely to happen by chance (binomial distribution test, p-value <2.2 × 10^−16^). Thus, only the 479 genes were analyzed in the following study, which were denoted as aging-DEGs.

The aging-DEGs and the AD-DEGs shared 21 genes, and all of them had consistent directions of correlations with age and expression changes (hypergeometric distribution test, p-value = 2.02 × 10^−11^). The pathway enrichment analysis showed that these aging- and AD-associated genes were also enriched in proteasome (p-value = 1.82 × 10^−4^) and RRAR signaling pathways (p-value = 3.70 × 10^−3^). Interestingly, more reserved gene pairs were found from AD patients with age ≤ 65 than AD patients with age > 65. In PWB-AD-249, the average numbers of reversed gene pairs identified from AD patients with age ≤ 65 and age > 65 were 529.64 ± 286.45 and 269.18 ± 228.31 (Wilcoxon rank sum test, p-value = 4.5 × 10^−3^), respectively. In PWB-AD-275, there were only three AD patients with age ≤ 65. Therefore, the phenomenon that the number of reserved gene pairs differed between the two groups was unobvious: the average numbers of reversed gene pairs identified from AD patients with age ≤ 65 and age > 65 were 282.52 ± 176.77 and 228.67 ± 163.95 (Wilcoxon rank sum test, p-value = 0.57), respectively. Actually, previous studies have compared the prevalence of a number of clinical features occurring in patients with early- and late-onset primary degenerative dementia of the Alzheimer type. The early-onset group demonstrated a greater prevalence of language disturbance, a disproportionate number of left-handers and a much shorter relative survival time than the late-onset group^39^.

These results showed that the occurrence of AD is closely related to aging, further suggesting that AD could be an excessive aging disease.

### Prediction performance of AD-associated cellular alterations in PWB

**Figure 4:**
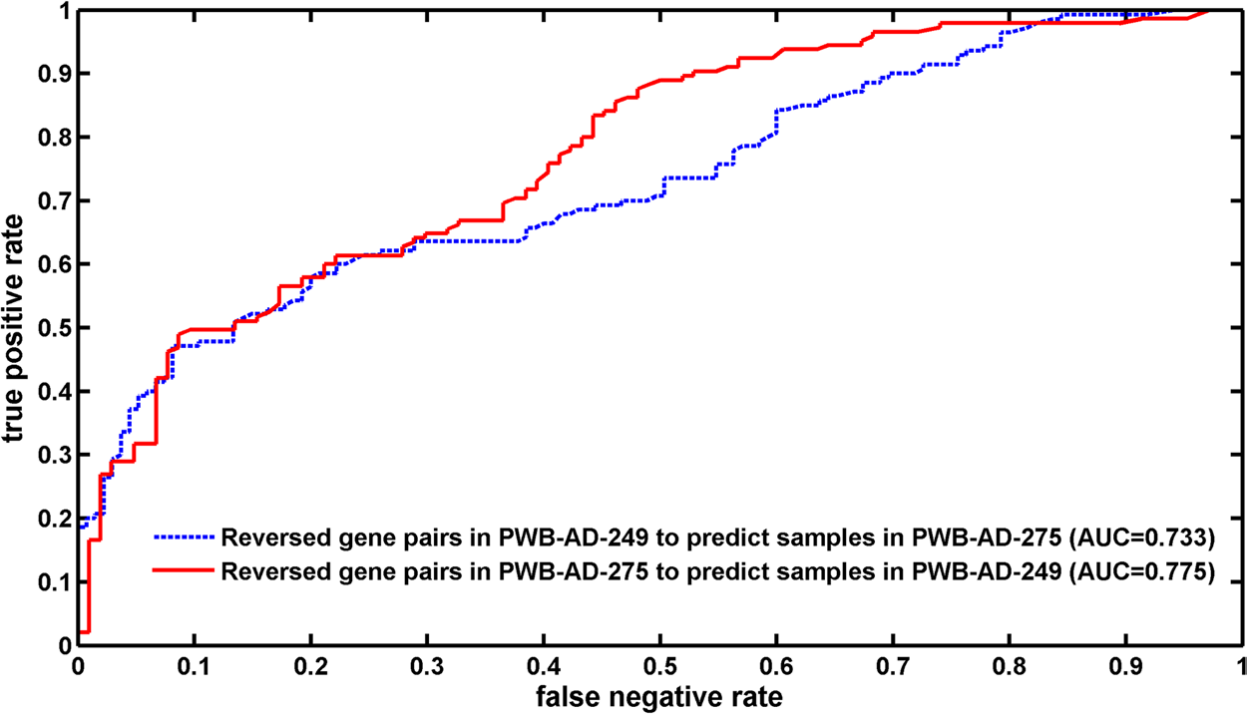
The prediction performance of reversed gene pairs.

One task of the blood-based test is to detect biomarkers for disease diagnostic workup. In this study, the detections of DEGs were based on gene pairs that have reversed REO patterns in AD patients compared with normal controls. These reversed gene pairs could be naturally used as diagnostic biomarkers for AD. The result showed that these reversed gene pairs had good prediction performance for classifying AD and normal samples: the 1,145 reversed gene pairs detected in PWB-AD-249 discriminated AD from the normal samples in PWB-AD-275 with an area under the curve (AUC) of 0.733, while the 1,249 reversed gene pairs detected in PWB-AD-275 discriminated AD from the normal samples in PWB-AD-249 with an AUC of 0.775 (Fig. 4). The prediction performances were better than the reported prediction performance by Nicola *et.al*. (AUC 0.729,^10^), which used a pathway based classification method based on the same data.

## Discussion

The concept of using blood such as peripheral blood cells as the source of information to detect disease-associated molecular alterations relies on the natural role of these cells in immune response to physiologic and pathologic changes^40^. In this study, we tried to detected AD-associated leukocyte cellular alterations from AD peripheral whole blood samples using the *Ref-REO* method^15^, which is based on within-sample REOs in purified leukocytes.

In the study, the number of PWB AD-DEGs identified by *Ref-REO* was relatively low, suggesting that the *Ref-REO* method had its disadvantages to some extent. Firstly, this method may fail to detect some disease-associated cellular alterations, as the reference pairs were required to have stable REO patterns in all purified leukocytes and in more than 95% of normal PWB samples, which could miss some AD-associated alterations. Secondly, the method of *Ref-REO* itself tended to detect DEGs with larger magnitude of cellular expression alterations, as greater expression alterations will have greater REO changes. Thus, genes with weak changes may be missed. Lastly, the *Ref-REO* method may not be able to detect those alterations occurring in leukocyte subtypes with limited proportions, as the signal could be easily covered by other leukocyte subtypes. However, it’s important to note that the PWB alterations identified by *Ref-REO* were closely associated with the development and progression of disease: the AD-DEGs were significantly enriched in AD-associated pathways, reproducible in brain tissue of AD patients and tended to interact with reported AD-associated biomarkers. Therefore, though low in quantity, the AD-DEGs identified by *Ref-REO* could be real expression alterations occurring in PWB leukocytes^15^.

Interestingly, our results showed that few AD-associated cellular alterations were enriched in PWB immune associated pathways or functional terms. In contrast, most of them were involved in aging-associated pathways or functional terms. For example, oxidative phosphorylation and mitochondrial have been reported to be closely associated with aging^41^. In AD blood samples, an increased neutrophil-lymphocyte ratio has also been reported as a function of age^42^. These results suggested that AD-associated cellular alterations in PWB might mainly reflect aging-associated alterations. Fortunately, such alterations could also be detected in the diagnostic workup of Alzheimer’s disease (AD), as AD is an excessive aging disease.
